# Effects of tongue tamers and customized bonded spurs as an early treatment of anterior open bite: a randomized clinical study

**DOI:** 10.1186/s12903-024-05389-x

**Published:** 2025-01-16

**Authors:** Safa B. Alawy, Shaimaa S. EL-Desouky, Ibrahim A. Kabbash, Shimaa M. Hadwa

**Affiliations:** 1 Department of Orthodontics, Faculty of Dentistry, Tanta University, Tanta, Egypt; 2 Department of Pediatric Dentistry, Oral Health, and Preventive Dentistry, Faculty of Dentistry, Tanta University, Tanta, 31527 Egypt; 3 Public Health & Community Medicine Department, Faculty of Medicine, Tanta University, Tanta, Egypt

**Keywords:** Tongue tamers, Open bite, Tongue thrusting, Bonded spurs

## Abstract

**Background:**

Anterior open bite is a challenging condition for pediatric dentists and orthodontists as it causes aesthetic, speech, feeding, and psychological problems; this emphasizes the need for early diagnosis and interception of this malocclusion.

**Aim:**

This study aimed to evaluate the effects of prefabricated metal-bonded tongue tamers and customized bonded spurs in the early treatment of anterior open bite.

**Materials and methods:**

A sample of seventy-five children aged 7–9 years were assigned into three groups in which anterior open bite was treated using tongue tamers (group-I), customized composite bonded spurs(group-II), and conventional fixed palatal cribs (group-III). Study model and cephalometric x-ray evaluations were done before and after a three-month follow-up. Data was gathered and statistically analyzed using ANOVA and Bonferroni tests.

**Results:**

Model overbite at central and lateral incisors was increased in all groups. The highest increase was found in group-I(3.16 ± 1.17, 2.81 ± 0.94)and group-III(3.08 ± 1.10,2.99 ± 1.00) though the difference was not statistically significant. Also, cephalometric overbite was non-significantly increased in all groups with a high increase in group-III(3.13 ± 1.10). Overjet decreased in all groups, with the greatest reduction observed in group-I(-2.58 ± 1.02 and -2.47 ± 0.80 in model and cephalometric respectively) and was significantly different from group-II using pairwise analysis. There was a decrease in SNA and ANB in all groups with more significant improvement in groups-I(-1.20 ± 0.88,-1.65 ± 0.74) and -III(-1.31 ± 0.92, -1.62 ± 0.75) than group-II(-0.63 ± 0.46, -0.90 ± 0.43). Moreover, FMA measurements decreased significantly in group-I (-2.6 ± 1.11). Regarding SNB measurement, there was a non-significant increase in all three groups with the highest increase in group-I(0.49 ± 0.48) followed by group-III(0.34 ± 0.63). The U1/FHP and L1/GoGn angles were non-significantly decreased in all tested groups with the highest decrease in group-I(-1.76 ± 1.00 and-2.54 ± 0.87 respectively).

**Conclusion:**

Early treatment of anterior open bite, along with tongue tamers' simplicity and aesthetics promoted the malocclusion correction and occlusal function restoration.

**Trial registration:**

ClinicalTrials.gov, NCT05792553, “Effects of Tongue Tamers as an Early Treatment of Anterior Open Bite”, Retrospectively registered: 31/03/2023.

## Introduction

Facial aesthetics are determined by a complex interplay of factors, including the shape and symmetry of the forehead, eyes, nose, lips, and underlying skeletal structures [1]. Also, establishing a stable and functional balance in the sagittal, vertical, and transverse dimensions is essential for achieving optimal facial aesthetics and overall attractiveness. Malocclusions and skeletal discrepancies in these planes can disrupt facial harmony, affecting not only dental function but also the proportionality and symmetry of the face [2]. Skeletal malocclusions, such as anterior open bite (AOB), disrupt this harmony by altering the vertical dimensions of the face and contributing to imbalances in both dental and skeletal structures [1]. An open bite is described as a localized absence of occlusion, typically observed between the maxillary and mandibular anterior teeth and it has a multifactorial etiology [3]. The prevalence of AOB in mixed dentition varies globally, influenced by genetic, environmental, and cultural factors; AOB affects approximately 17% of children and adolescents, with variations based on population and region [4]. From an etiologic standpoint, it might be categorized as a dental or skeletal open bite. Environmental factors like tongue thrusting or anterior tongue posture can produce a dental open-bite, whereas hereditary factors are primarily responsible for the etiology of skeletal open-bite [5].

Early intervention is crucial because of the negative AOB impact on a person's appearance, speech, nutrition, and psychology. Many therapeutic techniques were reported in the literature for interceptive treatment of anterior open-bite [6–8]. However, cessation and interception of any existing habit remains the primary therapeutic goal. Self-correction of dental AOB is possible in up to 80% of patients when the undesirable habit is eliminated during mixed dentition [9]. Banded spurs appliances were found to successfully address the anterior tongue posture by restraining the tongue from the anterior teeth, acting as a reminder to the patient, and sustaining long-term stability after treatment [10].

Recently, tongue tamers or bonded spurs was introduced in the market with obvious benefits including its small size, low price, aesthetics, lack of laboratory preparation, simplicity of installation, reduced chairside time, and can be used alongside fixed orthodontic therapy [11–13]. Few studies investigate the effect of tongue tamers as an interceptive method of AOB treatment [10, 13]. Lately, one study utilized a customized shark tooth-like spurs using compomer bonded to the lingual surface of anterior teeth to mimic the effect of the prefabricated tamers and concluded its beneficial impact on changing the tongue position [14].

By focusing on early treatment, practitioners can not only prevent the progression of AOB but also minimize its long-term effects on facial aesthetics and functionality. Early correction during the mixed dentition phase leverages growth potential, reducing the severity of skeletal discrepancies and restoring harmony to the facial profile [15]. Additionally, addressing AOB at a young age reduces the need for more invasive interventions in the future, supporting a stable, functional occlusion that positively impacts both dental health and their overall quality of life. So, this study aimed to evaluate the effectiveness of tongue tamers and customized bonded spurs compared to the conventional banded cribs as an early treatment of AOB. The null hypothesis (H_0_) assumed that there was no difference in the effective treatment of AOB in the early mixed dentition using tongue tamers or customized bonded spurs compared to the conventional banded cribs after a three-month follow-up.

## Material and methods

### Study setting and ethical consideration

A randomized controlled prospective clinical trial was conducted in the Outpatient Clinics of Pediatric Dentistry and Orthodontic Departments at the Faculty of Dentistry, Tanta University from March 2023 to October 2023. This study adheres to the CONSORT guidelines for randomized controlled trials. This trial was registered with the identifier NCT05792553 at ClinicalTrials.gov (Registered: 31/03/2023). The ethical committee (REC) of Tanta University's Faculty of Dentistry provided ethical permission for this study, with code (#R-ORTH-2-23-5) in accordance with the Helsinki Declaration of 1964 and its later revisions. Once parents signed a written informed consent, clinical treatment was started.

### Eligibility criteria

**Fig. 1:**
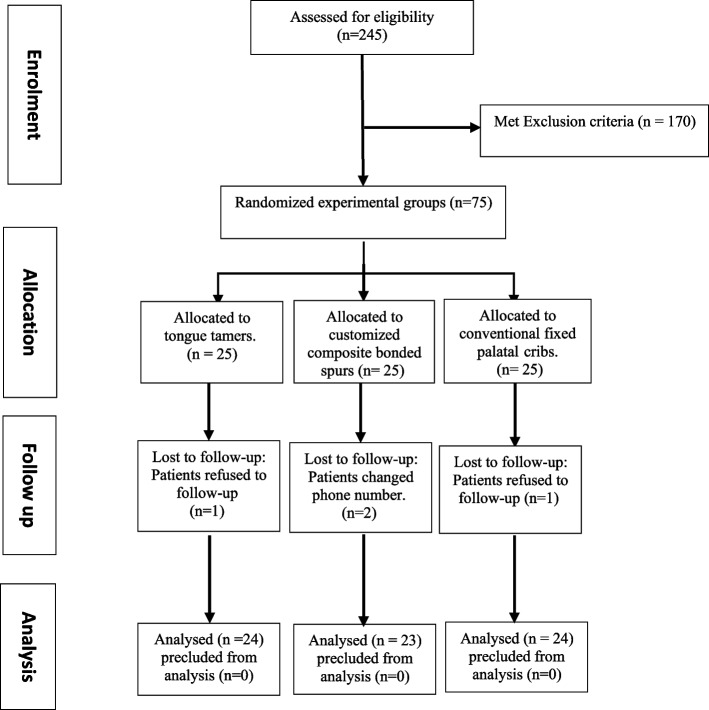
Flowchart outlining the patient’s randomization and assignment through this study

A total of 245 child patients aged 7–9 years were enrolled and assessed to meet the inclusion and exclusion criteria of the present study. Inclusion criteria were healthy cooperative children in early mixed dentition period with Angle Class I malocclusions, dental anterior open bite ≥ 1 mm [10, 16], and clinical signs of anterior tongue thrusting. Posterior crossbite, skeletal open-bite, crowding, tooth agenesis or loss of permanent teeth, and children with systemic diseases were the exclusion criteria [13]. Accordingly, one-hundred seventy child patients were precluded, leaving the final study sample of seventy-five children. A flow chart with enrollment, allocation, assessment, and sample size analysis is shown in Fig. 1.

### Sample size calculation and randomization

Using G Power version 3.1.9.2, the sample size was estimated based on the findings of Canuto et al., [13], and adopting a power of 80% (β = 0.20) to identify a standardized effect size in overbite (mm) (primary outcome) of 0.771 and a level of significance of 5% (α error accepted = 0.05). Twenty-two patients per group were the minimum needed sample size. To account for a 10% dropout rate, the sample size was expanded to 25 patients in each group (Total = 75 patients).

The process of randomization was conducted using the Research Randomizer software program (https://www.randomizer.org/) [17].

### Allocation concealment

An unbiased individual constructed a computer-generated randomized list, which was kept in an opaque sealed wrapper, to allocate those who fulfilled the inclusion criteria for any of the three groups.

### Group assignment

Seventy-five child patients were randomly allocated into three groups (25 children/group) according to different treatment modalities:

- **Group-I (experimental group)** (*n* = 25): Child patients were treated using prefabricated bonded tongue tamers (Matt Orthodontics, LLC, Chicago, USA).
- **Group-II (experimental group)** (*n* = 25): Child patients were treated using customized composite bonded spurs (3 M™ Filtek™ Z350 XT Universal Restorative, Minnesota, USA).
- **Group-III (positive control group)** (*n* = 25): Child patients were treated using conventional fixed palatal cribs (fabricated at the orthodontics Lab, Faculty of Dentistry, Tanta University)**.**

### Blinding

Due to the nature of the intervention, it was not possible to blind the patients or the orthodontist. The researcher and the statistician who evaluated the data were blinded.

### Clinical intervention

**Fig. 2:**
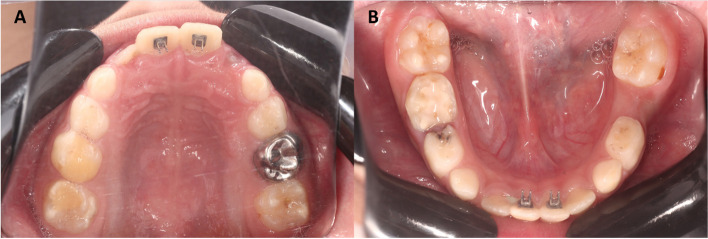
Prefabricated bonded tongue tamers were bonded on the upper (**A**) and lower (**B**) central incisors

**Fig. 3:**
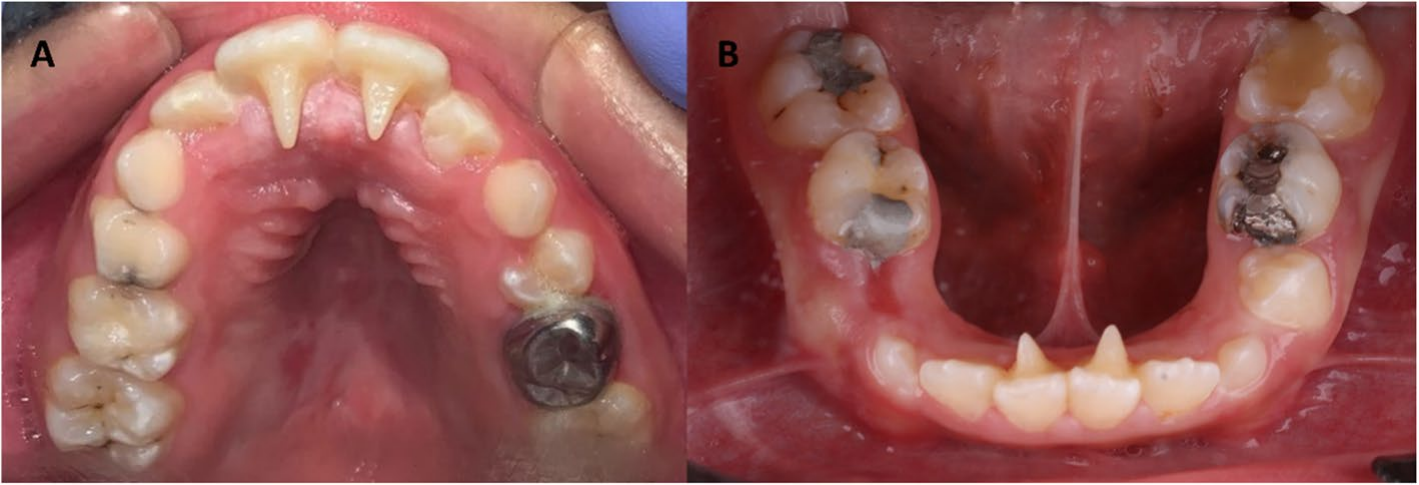
Customized composite bonded spurs were bonded on the upper (**A**) and lower (**B**) central incisors

Firstly, routine orthodontic records were taken including study model, panoramic radiograph, Lateral cephalometric radiograph (Orthophos XG DS/ceph, Sirona), and extra- & intra-oral photographs. In group-I, enamel surfaces of the palatal/lingual surfaces of the upper and lower central incisors were initially etched for 30 s using 37% phosphoric acid (T-Etchant, Nexobio, Korea). Then, tongue tamers were bonded using grēngloo™ (grēngloo™, two-color change adhesive, Ormco, California, USA) following the manufacturer’s instructions [13]. All tamers were bonded at the cervical third to prevent occlusal interferences upon the initial anterior open-bite correction (Fig. 2). In group-II, enamel surfaces of the palatal/lingual surfaces of the upper and lower central incisors were treated for 30 s with 37% phosphoric acid. Then, composite restoration was manipulated to make 3-mm spurs with sharp ends [14] (Fig. 3).

**Fig. 4:**
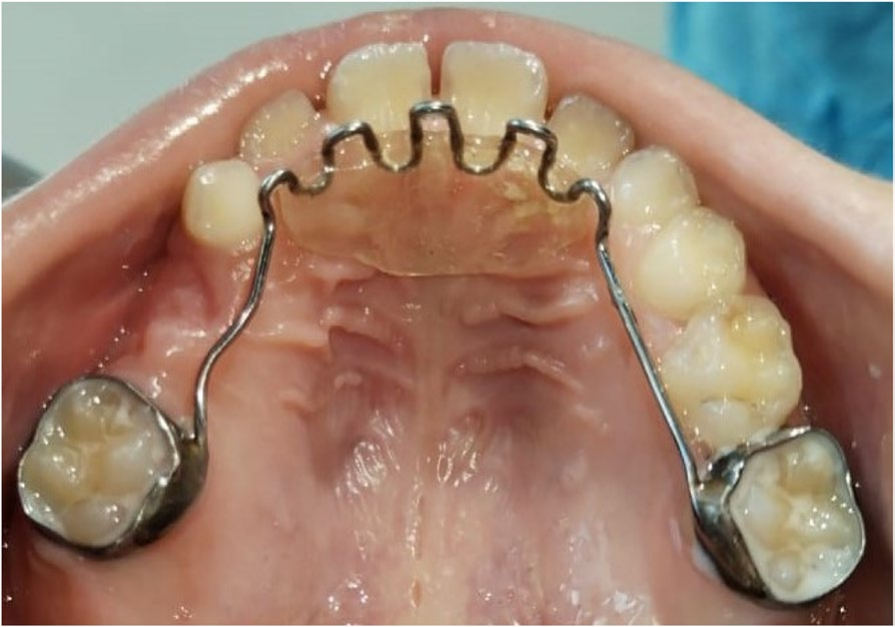
Conventional fixed palatal cribs after cementation

While in group-III, conventional fixed palatal cribs were prepared firstly, by band selection on the upper first permanent molars followed by taking an alginate impression (Cavex, Haarlem, Holland), then a palatal stainless-steel arch (Remanium®, Dentaurum, Ispringen, Germany) with cribs was designed utilizing 0.9 mm stainless-steel wire that soldered bilaterally to the bands (Matt Orthodontics, LLC, Chicago, USA) [11]. The spurs were constructed to be situated 3–4 mm behind the cingula of the maxillary incisors and pointed downward, ending behind the cingula of the lower incisors to assist a correct tongue posture. The spurs appliance was cemented with glass ionomer cement (Medicem, ProMedica Dental Material GmbH, Germany) (Fig. 4).

Child patients were instructed on certain precautions and post-operative recommendations about the used appliances for correcting tongue thrusting habits. These instructions included refraining from hard and sticky foods to preserve the integrity of the appliances, upholding meticulous oral hygiene with soft toothbrushes and mouth rinses to avoid plaque buildup around the appliances, and instantly reporting any discomfort or appliance-related difficulties. Also, they were instructed to press their tongue against the hard palate, bring the teeth into centric occlusion, and seal their lips to swallow. Moreover, parents should motivate their children to comply with the used appliances.

Follow-up visits were made biweekly during the initial month and monthly afterward to assess appliance performance and verify patient compliance.

### Model and cephalometric evaluation

After a three-month follow-up period, the same previously calibrated operator (first author) with 10 years of experience assessed all groups. Study models were obtained and compared to the pretreatment study models. Overbite was measured on the models on each anterior tooth pre- and post-treatment using a digital caliper (https://store.fut-electronics.com) [18]. The cephalometric variables including overbite, overjet, lower lip coverage of upper incisors, ANB°, SNA°, SNB°, FMA°, U1/FHP°, and L1/GoGn° were also assessed (Table 1). Facad ortho tracing (version 3.10, Sweden) was used to trace the lateral cephalometric radiographs [19]. Patients continued to receive treatment till at least 1 mm of positive vertical overlap was achieved in case their malocclusions were not corrected during the study period.

**Table 1:** Cephalometric variables measured in the study

Variable	Definition
SNA°	Angle created between Sella-Nasion (SN plane) to A point or Subspinale
SNB°	Angle created between Sella-Nasion (SN plane) to B point or supramentale
ANB°	Angle created between A, N, and B point
FMA°	Angle created between Frankfort Horizontal Plane (FHP) and Mandibular Plane (MP)
U1/FHP°	Angle made by the maxillary incisor's long axis and the FHP
L1/MP°	Angle made by the mandibular incisor's long axis to the MP
Overjet (mm)	Distance measured horizontally between the tips of the maxillary and mandibular central incisors
Overbite (mm)	Distance measured between the tips of the upper and lower central incisors in a vertical plane
Lower lip coverage of The upper incisors (mm)	Distance measured vertically between the upper incisal edge and the lower lip line perpendicular to FHP

### Statistical analysis

The obtained data was organized, tabulated, and analyzed using IBM's Statistical Package for the Social Sciences (SPSS) version 19. The variables were first tested for following the normal distribution by the Shapiro test. The range, mean, and standard deviation were computed for numerical values. The pre and post-within-group differences were tested using the paired t-test. The effect size of intervention was calculated as the difference between observation before intervention subtracted from that after intervention. The mean and standard deviation of the effect size were calculated, and the F-value (a one-way analysis of variance (ANOVA)) was used to assess the differences between groups. When the ANOVA test was found significant, the Bonferroni test was used for pairwise analysis. The significance level was adopted at p < 0.05. The Chi-square test was used to compare the distribution of gender among the studied groups.

The error of the method error was evaluated on randomly selected models and cephalometric radiographs (30% of the total sample) that were remeasured two weeks later. The data was subjected to a reliability coefficient to assess the intra-examiner reliability.

## Results

Table 2 displays all demographic data, including the distribution of enrolled children's ages and genders in each group. The distribution of enrolled children’s ages and genders in each group showed no significant difference. There was strong intra-examiner agreement, as indicated by kappa values for all assessments that were higher than 0.86. All patients were presented at three-month follow-ups except one patient in group-I and group-III rejected to come back for follow-up, also two patients in group-II were mislaid because of altering their telephone numbers. There were no significant variations in any of the analyzed models or cephalometric parameters between the three groups before treatment (Tables 3 and 4).

**Table 2:** Study participants' demographic distribution

**Demographic Characteristics**	**Group-I**	**Group-II**	**Group-III**		
Age				**F**	**P**
Range	8.0–9.0	7.5–9.6	7.8–9.6	**1.261**	**0.290**
Mean ± SD	8.93 ± 0.47	8.74 ± 0.57	8.72 ± 0.53		
Sex				***X***^***2***^	P
Males	12	11	13	**0081**	**0.777**
Females	13	14	12

**Table 3:** Comparison of model variables before and after treatment among studied groups

Model variables	Group I	Group II	Group III	F	p
**Central incisor overbite**					
Before	-3.32 ± 1.29	-3.78 ± 1.15	-3.89 ± 0.86	1.778	0.177
After	-0.16 ± 1.68	-1.24 ± 1.71	-0.81 ± 1.67		
*Effect size*	3.16 ± 1.17	2.54 ± 1.04	3.08 ± 1.10	2.174	0.122
***t (p)***	12.234**	11.752**	13.702**		
**Lateral incisor overbite**					
Before	-2.85 ± 1.25	-3.36 ± 1.15	-3.54 ± 0.94	2.465	0.093
After	-0.03 ± 1.42	-1.01 ± 1.53	-0.55 ± 1.56		
*Effect size*	2.81 ± 0.94	2.35 ± 0.89	2.99 ± 1.00	2.848	0.065
*** t (p)***	14.693**	12.610**	14.575**		
**Canine overbite**					
Before	-2.69 ± 0.107	-3.10 ± 1.11	-3.31 ± 0.97	2.106	0.129
After	-0.04 ± 1.20	-0.70 ± 1.52	-0.19 ± 1.51		
*Effect size*	2.6 ± 0.83	2.40 ± 0.94	3.12 ± 1.05	4.755^a^	0.008*
***t (p)***	14.081**	12.183**	14.529**		
**Model overjet**					
Before	5.35 ± 1.37	5.09 ± 0.87	5.08 ± 0.84	0.509	0.604
After	2.77 ± 0.62	3.43 ± 1.01	2.97 ± 0.79		
*Effect size*	-2.58 ± 1.02	-1.66 ± 0.64	-2.12 ± 0.81	7.047^b^	0.002*
***t (p)***	12.375**	12.333**	12.733**		

^*^Significant

^**^p < 0.001

^a^Group-III significantly different from group-I and -II using pairwise comparison

^b^Group-I significantly different from group-II using pairwise comparison

**Table 4:** Comparison of cephalometric variables before and after treatment among studied groups

Variables	Group I	Group II	Group III	F	P
Overbite					
Before	-3.23 ± 1.30	-3.74 ± 1.15	-3.89 ± 0.85	2.274	0.111
After	-0.12 ± 1.72	-1.24 ± 1.73	-0.76 ± 1.70		
*Effect size*	3.11 ± 1.16	2.50 ± 1.03	3.13 ± 1.10	2.454	0.093
*t (p)*	13.162 **	11.610**	13.938**		
SNA					
Before	83.52 ± 1.19	82.76 ± 2.63	83.17 ± 2.16	0.085	0.919
After	82.32 ± 0.60	82.13 ± 2.78	81.86 ± 1.85		
*Effect size*	-1.20 ± 0.88	-0.63 ± 0.46	-1.31 ± 0.92	4.959^a^	0.010*
*t (p)*	6.706**	6.663**	6.940**		
SNB					
Before	78.90 ± 0.85	78.16 ± 2.69	78.89 ± 2.10	1.025	0.364
After	79.9 ± 0.79	78.43 ± 2.73	79.23 ± 1.97		
*Effect size*	0.49 ± 0.48	0.23 ± 0.28	0.34 ± 0.63	1.756	0.180
*t (p)*	5.038**	5.426**	2.653***		
ANB					
Before	4.65 ± 1.12	4.62 ± 1.61	4.50 ± 1.07	0.786	0.460
After	3.00 ± 0.97	3.72 ± 1.55	2.88 ± 0.70		
*Effect size*	-1.65 ± 0.74	-0.90 ± 0.43	-1.62 ± 0.75	9.776^a^	< 0.001*
*t (p)*	10.930**	9.938**	10.586**		
FMA					
Before	25.75 ± 2.47	26.70 ± 2.29	26.94 ± 2.01	1.947	0.146
After	23.10 ± 1.39	25.67 ± 2.11	25.72 ± 2.21		
*Effect size*	-2.6 ± 1.11	-1.03 ± 0.63	-1.22 ± 0.97	3.181^b^	0.001*
*t (p)*	8.393**	7.846**	6.192**		
U1/FHP					
Before	113.69 ± 1.13	112.26 ± 2.89	113.12 ± 2.21	2.525	0.088
After	111.93 ± 0.73	110.93 ± 2.45	111.51 ± 1.81		
*Effect size*	-1.76 ± 1.00	-1.34 ± 0.74	-1.62 ± 0.86	1.384	0.258
*t (p)*	8.577**	8.727**	9.166**		
L1/GoGn					
Before	98.84 ± 1.44	98.56 ± 2.11	97.84 ± 2.27	1.648	0.200
After	96.30 ± 1.47	96.45 ± 2.04	96.09 ± 1.98		
*Effect size*	-2.54 ± 0.87	-2.11 ± 1.29	-1.75 ± 1.10	3.100	0.051
*t (p)*	14.229**	7.847**	7.806**		
Overjet					
Before	5.24 ± 1.14	5.06 ± 0.85	4.98 ± 0.88	0.510	0.603
After	2.78 ± 0.65	3.49 ± 1.02	2.92 ± 0.77		
*Effect size*	-2.47 ± 0.80	-1.57 ± 0.70	-2.05 ± 0.79	8.083^b^	0.001*
*t (p)*	15.126**	10.781**	12.781**		
Lower lip coverage of the upper incisors					
Before	-1.88 ± 0.97	-1.99 ± 0.77	-1.96 ± 0.60	0.095	0.910
After	1.78 ± 0.91	1.65 ± 0.61	1.74 ± 0.51	0.219	0.804
*Effect size*	3.99 ± 1.14	3.64 ± 0.95	3.70 ± 1.00	0.024	0.976
*t (p)*	15.711**	18.307**	18.174**		

^*^Significant

^**^p < 0.001

^***^p = 0.014

^a^Group II significantly different from groups I and III using pairwise comparison

^b^Group I significantly different from group II using pairwise comparison

### Model analysis

Overbite measurements on the model at the central and lateral incisors were increased in all treatment groups with a high increase found in group-I and -III with non-significant statistical differences (*p* = 0.122, *p* = 0.065 respectively) (Table 3). While there was a statistically significant increase in canine overbite measurement on the model among different groups with the highest increase reported in group-III. Using pairwise analysis, group-III was significantly different in canine overbite measurement from groups-I and -II. Regarding intragroup findings, all groups showed a statistically significant increase in the initial overbite measurements (*p* < 0.001). Concerning model overjet measurement, the highest decrease (-2.58 ± 1.02) was recorded in group-I with statistically significant differences among treatment groups, also using pairwise analysis, group-I was significantly different from group-II in model overjet measurements. Using paired t-test for intragroup difference, all groups recorded a statistically significant decrease in the initial overjet values (*p* < 0.001).

### Cephalometric analysis

As shown in Table 4, overbite was increased in all treatment groups with a high increase reported in group-I and group-III (3.11 ± 1.16, 3.13 ± 1.10 respectively) with a non-significant statistical difference (*p* = 0.093). Regarding the intragroup results, all groups revealed a statistically significant increase in the initial values of cephalometric overbite (*p* < 0.001). In addition, there was a decrease in SNA, ANB, FMA, and overjet measurements with a statistically significant difference among different groups (*p* < 0.05). Also, the intragroup comparison using paired t-test showed a statistically significant decrease in the initial values of SNA, ANB, FMA, and overjet within each treatment group (*p* < 0.001). Using pairwise analysis, group-II was significantly different in SNA, and ANB measurements from groups-I and -III moreover, group-I was significantly different from group-II in overjet and FMA measurements. Regarding SNB measurement, the highest increase (0.49 ± 0.48) was reported in group-I with non-significant statistical difference among different groups (*p* = 0.180) while a statistically significant increase was found in its initial value within each group (*p* < 0.001). Concerning the measurements of U1/FHP and L1/GoGn angles, the highest decrease was noted in group-I (-1.76 ± 1.00 and -2.54 ± 0.87 respectively) with non-significant statistical differences among treatment groups (*p* = 0.258, *p* = 0.051 respectively) and statistically significant differences within each treatment group (*p* < 0.001). The amount of lower lip coverage of the upper incisors increased in all groups, with group I showing the highest increase, followed by group III, then group II (3.99 ± 1.14, 3.70 ± 1.00, and 3.64 ± 0.95mm, respectively) with a non-significant statistical difference (*p* = 0.976). The intragroup comparison revealed a statistically significant increase in the initial values of the amount of lower lip coverage of the upper incisors (*p* < 0.001).

## Discussion

The stomatognathic system's balance is essential to occlusal physiology and craniofacial development. An AOB can be categorized as functional, skeletal, dentoalveolar, or a combination, based on the underlying cause. Skeletal open bites result from excessive vertical growth of the dentoalveolar complex, particularly in the posterior molar region. Patients with this condition often exhibit an increased lower facial height relative to the upper facial height, commonly referred to as long-face syndrome [20]. In contrast, anterior dental open bites are typically associated with a normal craniofacial pattern, proclined and undererupted anterior teeth, and habits like thumb sucking or tongue thrusting [21]. Also, anterior dental open bite has a significant impairment to both appearance and functionality (mastication and speech) because of possible changes to the skeleton and teeth [22]. Furthermore, children may have psychological problems, making early intervention absolutely necessary [23]. Correcting anterior dental open bite remains a challenge for both pediatric dentists and orthodontists as it should be diagnosed and treated early in the mixed dentition phase. By removing the etiological cause, malocclusion may be corrected leading to improvements in the occlusal, functional, and aesthetic domains [24]. This study aimed to assess the effectiveness of tongue tamers and customized bonded spurs compared to the conventional banded cribs as an early treatment of anterior dental open bite.

Prefabricated or customized bonded spurs were used in this study as they help alter the anterior tongue-resting position preventing it from interposing between incisors. Moreover, these appliances are simple to apply and remove, eliminate the banding procedures and laboratory phase, are inexpensive, suitable for both arches with no visual interference, and can be exploited in combination with fixed orthodontic appliances [25]. Also, in this study spurs were simply attached to the upper and lower central incisors, adding more simplicity to the appliance.

The age range of the present study was 7–9 years, which is an inter-transitional period with a quiescent development pattern. Consequently, the use of fixed or removable devices was permissible without compromising individual growth [26]. Moreover, it has been claimed that intervention for the AOB is mandatory at this stage since interrupting the undesirable habit alone is no longer ensured for self-correction, despite it may still happen [27].

The results of this study accepted the null hypothesis because a non-significant statistical difference was found between the treatment groups concerning overbite measurements on cephalometric and model at the central and lateral incisors at a three-month follow-up period, while a significant difference was detected in canine overbite measurement on the model among different groups (null hypothesis was partially rejected).

Overbite measurements on the model at the central and lateral incisors were increased in all treatment groups with a high increase found in tongue tamers and conventional cribs groups with a non-significant statistical difference; this could be ascribed to retrusion of the maxillary and mandibular incisors, eradication of the tongue contact and clinical enhancement in lip posture [13]. Also, there was a significant statistical increase in canine overbite measurement on the model among different groups with the highest increase reported in the conventional cribs group. This may be attributed to the more coverage provided by conventional cribs against tongue pressure extending to the canine region, in contrast to tamers which bonded only to the central incisors.

Cephalometric overbite findings were consistent with the model measurements which showed an increase in all treatment groups with a high increase reported in the tongue tamers and conventional cribs groups with no significant difference among all treatment groups. This improvement in overbite concurred with Rossato et al., [8] who found that the bonded spurs group resulted in a reduction in the pretreatment open bite from -4.03 mm to -0.94 mm. Also, it coincided with Pedrin et al. [28], who found an improvement in overbite from -4.01 to 1.02 mm in the treated group (removable palatal crib and high-pull chin cup therapy). On the other hand, Leite et al., [11] disagreed with the present study results since they reported that the overbite improvement in the conventional crib group was the largest in comparison to the spur and control group (3.95, 3.07, and 2.33 mm, respectively). In this study, the increase in cephalometric overbite was statistically non-significant among all treatment groups; this was contrasted to Canuto et al., [13] who found significantly greater overbite increases (4.26 mm) in the tested groups (bonded lingual spurs and conventional lingual spurs) than in the control group. Also, Cassis et al. [10], reported a significantly greater overbite increase (5.23 mm) in the treated group (lingual bonded spurs and high-pull chin cup) than in the control group. This conflict may be accredited to the short treatment period of the present study also, the selection of untreated patients as a control group in Canuto et al., [13] and Cassis et al., [10] studies.

In the current study, there was a decrease in SNA, ANB, and FMA measurements with a significant statistical difference among different groups. This disagreed with Leite et al., [11] who reported no statistically significant difference in most cephalometric variables analyzed (SNA, ANB, SNB, IMPA, SnGoGn, 1PP, and nasolabial angle) between fixed palatal crib & lingual bonded spur after twelve months of follow-up. Also, these results contrasted with Rossato et al., [8] who found a non-significant decrease in SNA, ANB, and FMA among the studied groups (bonded spurs, fixed palatal crib, chin cup, and removable palatal crib). This may be attributed to the differences in follow-up periods and AOB severity of the selected patients.

Regarding SNB measurement, the highest increase was reported in the tongue tamers group with non-significant statistical differences among different groups; this coincided with Rossato et al., [8] who revealed a non-significant increase in SNB measurement (0.18) in the bonded spurs group. The SNB increase reflects the anti-clockwise rotation of the mandible, which may contribute to AOB closure [29].

Concerning the measurements of U1/FHP and L1/GoGn angles in this study, the highest decrease was noted in the tongue tamers group with non-statistically significant differences among treatment groups; this may be clarified by the fact that in some patients, the overbite was corrected by extrusion alone, without inclination. This agreed with Leite et al., [11] who revealed minor changes in 1/PP and IMPA without statistical significance between the fixed palatal crib and bonded lingual spur after twelve months of follow-up.

Regarding overjet measurement, the highest decrease (-2.47) was recorded in the tongue tamers group with statistically significant differences among treatment groups; possible connections include the elimination of tongue interposition or disruption of oral habits, maturation of the face, and the attainment of muscular equilibrium [30]. Moreover, the greater palatal tipping of the maxillary incisors to the mandibular incisors could lead to a greater decrease in overjet [13]. This agreed with Canuto et al., [13] who found a significantly greater decrease in overjet in the bonded lingual spurs group compared to the control group. On the other hand, this disagreed with Bublitz et al., [30] who reported a non-significant overjet decrease (-0.27) in the lingual spurs group compared to the chin cup group (0.12) (*p* = 0.2). The conventional fixed palatal cribs in this study showed a decrease in overjet measurements (-2.05 ± 0.79); this was contrasted to the results of Torres et al. [16], study in which overjet was increased by 0.4 mm in the fixed palatal cribs with the chin cup group. This conflict arises because, when a fixed crib is used, the lingual pressure produced by the perioral muscles upon the mandibular incisors, particularly during swallowing, is not counterbalanced by the tongue pressure because it permanently stays in a backward position, and this alteration in the muscle equilibrium may lead to the inclination changes in mandibular incisors [16].

In the current study, the mean lower lip coverage of the upper incisors was improved from -1.88 ± 0.97 to 1.78 ± 0.91 mm in group I, from -1.99 ± 0.77 to 1.65 ± 0.61 mm in group II, and from -1.96 ± 0.60 to 1.74 ± 0.51 mm in group III. This improvement in the vertical lower lip position reflects the improvement in the anterior lip seal and may be attributed to the increase in overbite and the decrease in overjet achieved in different study groups.

The limitation of this study was the absence of an untreated control group, a problem noted in numerous studies [31]. In these types of studies, it may be unethical to leave the control group untreated with unnecessary radiation exposure and keep them without intervention ignoring their demand for quick treatment [32]. Additional limitations include the short follow-up and the lack of detailed assessment and consideration of tongue thrusting as a contributing factor to anterior dental open bites which may limit the generalizability of the study. Future research should explore the influence of tongue posture and function to provide a more comprehensive understanding of the condition. Also, more studies are required to investigate the effects of prolonged treatment periods and treatment stability.

Based on the present study findings of the lack of significant differences in overbite changes, it can be suggested that tongue tamers and customized bonded spurs tend to be more convenient in clinical practices as they eliminate the banding and laboratory phases. Also, both appliances were well tolerated by children during chewing, eating, and speech. Although the customized bonded spurs were fixed, there was recurrent debonding and decreased sharpness, a factor that should be respected at the time of appliance choice [13]. Also, the possibility that the spurs is detached and swallowed alongside irritation of the tip of the tongue should be considered.

## Conclusion

- All tested appliances resulted in a high overbite increase during the early treatment of anterior open bite.
- Prefabricated tongue tamers showed greater improvement in the positioning of the incisors and subsequently in the correction of overjet compared to customized bonded spurs over a three-month follow-up period.
- Tongue tamers offer an effective early treatment of anterior open bite that promotes malocclusion correction and occlusal function restoration.

## Data Availability

The datasets used and/or analyzed in the present study can be obtained from the corresponding author upon reasonable request.
